# DNA methylation age is not affected in psoriatic skin tissue

**DOI:** 10.1186/s13148-018-0584-y

**Published:** 2018-12-27

**Authors:** Changbing Shen, Leilei Wen, Randy Ko, Jing Gao, Xue Shen, Xianbo Zuo, Liangdan Sun, Yi-Hsiang Hsu, Xuejun Zhang, Yong Cui, Meng Wang, Fusheng Zhou

**Affiliations:** 1 Institute and Department of Dermatology, The First Affiliated Hospital, Anhui Medical University, Hefei, 230032 Anhui China; 2 Department of Dermatology, China-Japan Friendship Hospital, Beijing, 100029 China; 3 Graduate School, Peking Union Medical College and Chinese Academy of Medical Sciences, Beijing, 100730 China; 4 Department of Biochemistry, University of New Mexico, Albuquerque, NM 87131 USA; 5 Department of Dermatology, The Second Affiliated Hospital, Anhui Medical University, Hefei, 230601 Anhui China; 6 Hebrew SeniorLife Institute for Aging Research and Harvard Medical School, Boston, MA 02131 USA; 7 Molecular and Integrative Physiological Sciences, Harvard T.H. Chan School of Public Health, Boston, MA 02115 USA; 8 Broad Institute of MIT and Harvard, Cambridge, MA 02142 USA; 9 Institute and Department of Dermatology, Huashan Hospital of Fudan University, Shanghai, 200040 China; 10 Department of Environmental Health, Harvard T.H. Chan School of Public Health, Boston, MA 02115 USA

**Keywords:** Psoriasis, DNA methylation age, Skin tissue, Chinese Han population

## Abstract

**Background:**

Psoriasis (Ps) is a common chronic inflammatory skin disease. The keratinocytes of psoriatic skin defy normal apoptosis and exhibit active cell proliferation. Aberrant DNA methylation (DNAm) has been suggested relevant through regulating the expression of Ps susceptibility genes. However, it is unclear whether the biological age inferred from DNA methylome is affected.

**Results:**

To address the above issue, we applied a recently developed methylation clock model to our Chinese Han population dataset, which includes DNAm data of 114 involved psoriatic skin tissues (PP) and 41 uninvolved psoriatic skin tissues (PN) from Ps patients, and 62 normal skin tissues (NN) from health controls. We first confirmed the applicability of the clock in PN and NN. We then showed that PP samples have largely unchanged DNAm age, and that no association was observed between available clinical features and DNAm age acceleration. Examination of genome-wide CpGs yielded age-associated CpGs with concordant age-association coefficients among the three groups, which was also supported by an external dataset. We also interestingly observed two clock CpGs differentially methylated between PP and PN.

**Conclusions:**

Overall, our results suggest no significant alteration in DNAm age in PN and PP. Therefore, the increase in keratinocyte proliferation and alteration in DNAm caused by Ps may not affect the biological age of psoriatic skin tissue.

**Electronic supplementary material:**

The online version of this article (10.1186/s13148-018-0584-y) contains supplementary material, which is available to authorized users.

## Introduction

Psoriasis (Ps) is a chronic autoimmune inflammatory skin disease and affected by a complex interplay between the immunologic factors, Ps-associated susceptibility loci and various environmental factors [1–3]. This disease is characterized by abnormal growth, differentiation, and apoptosis of keratinocytes [4]. In normal skin apoptosis offsets keratinocyte proliferation and regulates the formation of stratum corneum. However, psoriatic keratinocytes exhibit an enhanced ability to resist apoptosis. This is a key potential step during the pathogenesis of Ps [5]. Whereas such resistance leads to the abnormal and rapid proliferation of keratinocytes, it remains unclear whether it causes acceleration or deceleration in the biological aging process of skin tissue.

Importantly, the age of onset is a key clinical character for classifying Ps subtypes [6]: patients are classified as early stage (or type I) if their onset ages are < 40 years old or late stage (or type II) if otherwise. In a UK cohort, the onset age of Ps incidence is bimodally distributed, with a clear separation between early and late stage patients [7]. The two subtypes have differences in certain aspects. For example, only early but not late stage patients are strongly associated with the HLA-C*06:02 allele in a case-control study [8]. Moreover, patients from the two stages are unevenly distributed across the three Ps subgroups classified based on DNA methylation (DNAm) [9]. These findings indicate the existence of an age effect during the occurrence and progression of Ps.

Ps is associated with change in DNAm levels at various CpG sites, which regulate the expression of genes at key processes related to Ps progression [10]. On the other hand, DNAm can record the biological age of an organism. Numerous CpGs throughout the genome can increase or decrease their methylation levels during the aging process, a pattern that can be observed ubiquitously across tissues and species [11–19]. The prevalence of age-associated CpGs has led to the development of epigenetic aging markers (or DNAm clocks) that use the combination of tens to a few hundreds of CpGs to predict age [17, 18, 20–25]. These DNAm clocks have been proven competent in predicting age in various cell/tissue types with reasonable accuracy [18, 22, 23]. Beyond accurately predicting age, DNAm clocks can identify the change in lifespan that are caused by genetic and environmental interventions [20–22] and indicate the acceleration of biological age caused by pathological conditions [26–28]. These suggest the usefulness of these clocks in understanding the impact of chronic diseases on lifespan.

Given the involvement of age and DNAm in Ps progression, and the active proliferation of keratinocytes, here we asked whether Ps skin tissues have anomalous DNAm ages in the Chinese Han population. To test this, we applied a DNAm clock model to a dataset from a previous epigenome-wide association study [29], which includes 114 involved psoriatic skin tissues (PP) and 41 uninvolved psoriatic skin tissues (PN) from Ps patients, and 62 normal skin tissues (NN) from health controls (Table 1).

**Table 1:** Detailed characteristics of study samples

Sample characteristics	Psoriasis patients		Health controls	*P* value
	PP	PN	NN	
Number	114	41	62	–
Sex				
Male	66	26	25	0.032
Female	48	15	37	
Age				
Range	10.0–76.0	15.0–73.0	15.0–75.0	0.91
Mean ± SD	37.3 ± 14.4	37.6 ± 15.5	40.8 ± 14.6	
BMI				
Range	16.6–32.8	17.3–28.3	16.8–29.1	0.99
Mean ± SD	22.8 ± 2.9	22.4 ± 2.3	22.3 ± 2.8	
PASI				
Range	0.6–16.0	–	–	–
Mean ± SD	4.1 ± 3.1	–	–	
Smoking status				
Current	24	8	15	0.038
Former	13	3	8	
Never	77	30	39	

## Results and discussion

**Fig. 1:**
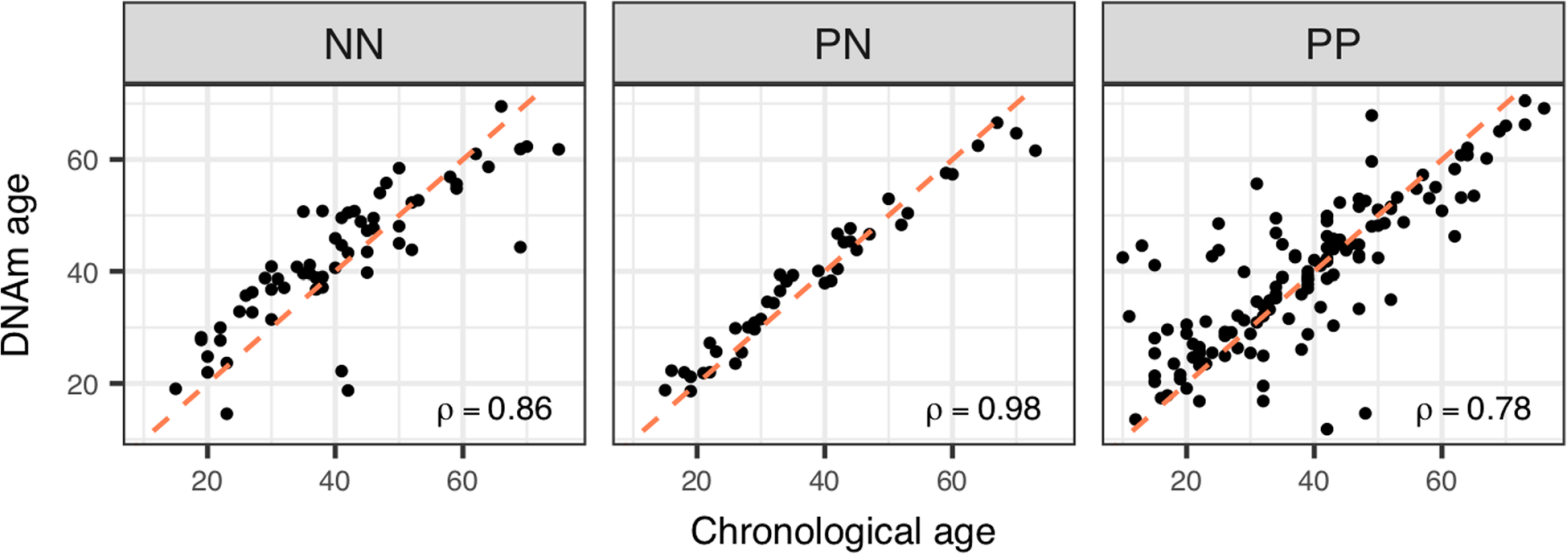
Scatter plots showing the correlation between DNAm age and chronological age for NN, PN, and PP (Spearman’s ρ of NN, PN, and PP are 0.86, 0.98, and 0.78 respectively, *P* < 2.2E-16. The red dashed lines denote where DNAm age and chronological age are equal)

**Fig. 2:**
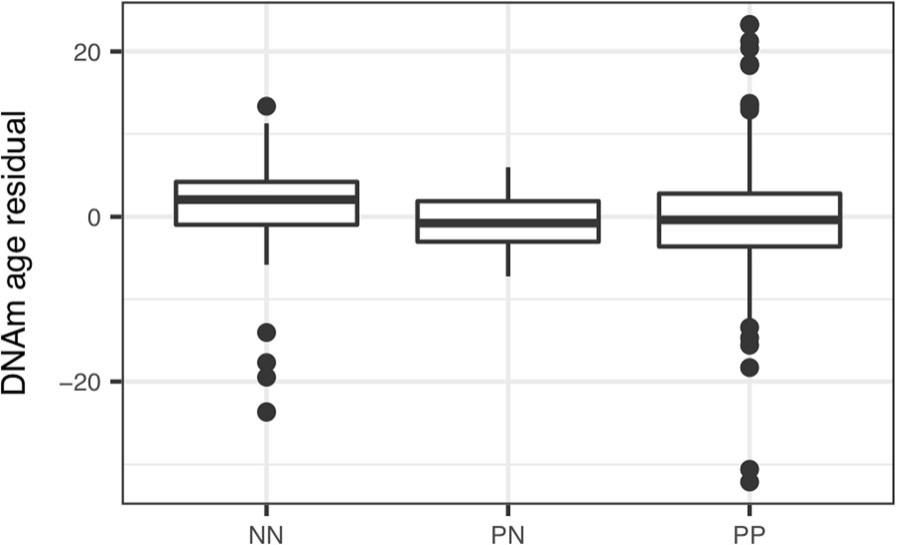
DNAm age residuals of NN, PN, and PP (*t* test compared to 0, *P* values are 0.28, 0.15, and 0.78, respectively)

Here, we focused on the multi-tissue DNAm age clock developed by Horvath [18, 25]. We first examined normal skin tissues (NN and PN) to address the accuracy of this age predictor on our methylation dataset. Indeed, we observed a strong positive correlation between the estimated DNAm age and chronological age for both NN and PN (Fig. 1, Spearman’s ρ ≥ 0.86, *P* < 2.2E-16). Moreover, the age acceleration residuals, which measure the increase/decrease in DNAm age relative to chronological age, are near zero for both NN and PN (Fig. 2, *t* test *P* > 0.15). Therefore, these suggest that the methylation age clock model works reasonably well in NN and that the DNAm age of PN is unchanged. We then examined the DNAm data of PP. A strong correlation between DNAm age and chronological age was also observed (Fig. 1, ρ = 0.78, *P* < 2.2E-16), although somewhat lower than that from NN and PN. Moreover, the age acceleration residual of PP also showed no significant difference from zero (Fig. 2, *t* test, *P* = 0.78), or compared to NN and PN (*P* > 0.3). The different correlations between DNAm age and chronological age in the three groups may be affected by some external reasons, for example, previous studies have shown that numerous conditions such as obesity [26], Huntington’s disease [30], race [31], and calorie restriction [21] could somehow affect DNAm age. We further specifically examined the 41 paired PP and PN samples and again did not observed significant difference in age acceleration residual (paired *t* test, *P* = 0.51). These data suggest that Ps may not alter the DNAm age of PP.

To explore the potential impact of clinical features on DNAm age acceleration across samples, we applied a multi-linear model to associate age acceleration residual with available clinical features. These include age, body mass index (BMI), gender, smoking status (current, former, and never), and Ps area and severity index (PASI) score. As a result, we observed no significant association between clinical features and age acceleration residual in all skin groups (Table 2).

**Table 2:** Multi-variable linear regression coefficients of clinical features with DNAm age residuals for NN, PN, and PP

Statistics	Estimate	Std. error	*t* value	Pr (>|*t*|)
1) Model for NN: age acceleration residual~age + BMI + gender + smoking status				
Intercept*	− 0.764	7.678	− 0.100	9.21E-01
Age	− 0.038	0.068	− 0.556	5.80E-01
BMI	0.200	0.352	0.569	5.72E-01
Gender (female vs. male)	− 0.830	1.863	− 0.446	6.58E-01
Smoking status (current vs. former)	−1.789	3.067	− 0.583	5.62E-01
Smoking status (current vs. never)	− 0.803	2.123	− 0.378	7.07E-01
2) Model for PN: age acceleration residual~age + BMI + gender + smoking status				
Intercept	− 8.309	4.561	− 1.822	7.70E-02
Age	0.138	0.034	4.074	2.52E-04
BMI	0.125	0.206	0.607	5.48E-01
Gender (female vs. male)	− 1.688	0.975	− 1.732	9.21E-02
Smoking status (current vs. former)	− 2.745	1.865	−1.472	1.50E-01
Smoking status (current vs. never)	0.536	1.222	0.438	6.64E-01
3) Model for PP: age acceleration residual~age + BMI + gender + smoking status + PASI				
Intercept	6.266	6.644	0.943	3.47E-01
Age	− 0.032	0.053	− 0.605	5.47E-01
BMI	− 0.196	0.286	− 0.685	4.95E-01
Gender (female vs. male)	− 1.173	1.656	− 0.708	4.80E-01
PASI scores	− 0.255	0.198	− 1.289	2.00E-01
Smoking status (current vs. former)	3.780	2.567	1.472	1.43E-01
Smoking status (current vs. never)	0.573	2.019	0.284	7.77E-01

*Intercept is the constant term of a regression model. Here, it denotes the corresponding value of age acceleration residual when all the independent variables are 0

**Fig. 3:**
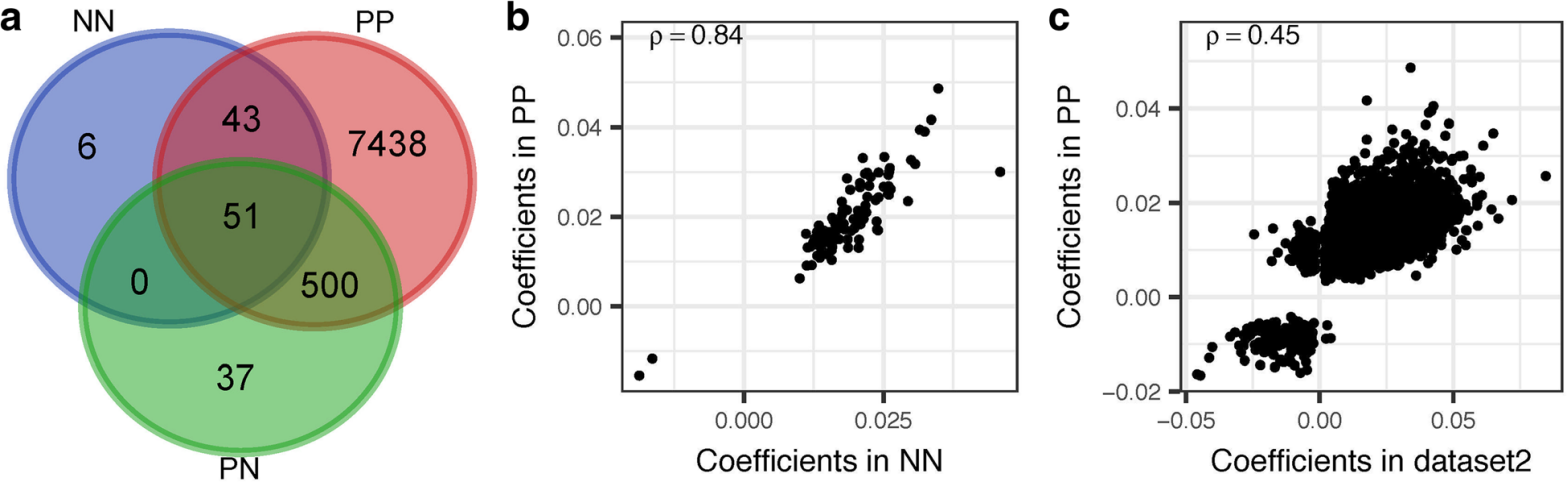
Comparison of age-associated CpGs among groups and with external dataset. **a** The overlap of age-associated CpGs across the NN, PN, and PP groups. **b** For the 94 overlapped age-associated CpGs between NN and PP, their coefficients are strongly positively correlated (Spearman’s ρ = 0.84, *P* < 2.2E-16). **c** For the overlapped age-associated CpGs between an additional epidermis dataset and PP, their coefficients are also significantly positively correlated (Spearman’s ρ = 0.45, *P* < 2.2E-16)

We observed 100, 588, and 8032 CpGs, respectively, in NN, PN, and PP that are significantly associated with age (false discovery rate (FDR) < 0.05, Additional file 1: Table S1, Additional file 2: Table S2, and Additional file 3: Table S3). Interestingly, almost all of these significant CpGs (95, 585, and 7765 CpGs in NN, PN, and PP, respectively) undergo hypermethylation during aging. Also, almost all of these CpGs in NN (94.0%) and PN (93.7%) remain age-associated in PP, while 51 CpGs are shared across the three groups (Fig. 3a). All these overlaps are significantly higher than by chance (hypergeometric test *P* < 2.2E-16). Moreover, the shared CpGs have largely consistent age-association coefficients across comparisons (ρ > = 0.75, Fig. 3b, Additional file 4: Figure S1A and S1B). Despite the consistence in coefficients, the discrepancy in numbers of age-associated CpGs across groups seems intriguing. To address whether this is genuine or caused by factors such as sample size and the range of age, we considered specifically the 41 individuals with paired PN and PP samples appears suitable to account for these effects. As a result, we observed 588 and 325 CpGs (Additional file 5: Table S4) associated with age (FDR < 0.05) in these paired PN and PP samples, respectively, which are rather comparable numbers. Therefore, the discrepancies in numbers of age-associated CpGs across groups are rather a reflection of difference in statistical power.

To further assess these age-associated CpGs, we re-analyzed a study of 108 healthy epidermis methylomes measured under the same platform [32]. We observed that the vast majority of age-associated CpGs (> 77.5%) are also significant in this dataset, and that the age-association coefficients are significantly positively correlated (ρ ≥ 0.45, Fig. 3c, Additional file 4: Figure S1C and S1D). These results support the consistency across the three categories of skin tissue samples and with external skin data.

Our previous analyses identified 426 and 1514 CpGs differently methylated between PP and NN, and between PP and PN, respectively, with 264 shared between the two comparisons [29]. We then asked how many Ps-specific CpGs are also age-associated. Among the 8032 age-associated CpGs observed in PP, we identified 39 (Additional file 6: Table S5) and 1 (cg11426075) CpGs also differentially methylated between PP and PN, and between PP and NN, respectively. The age- and Ps-associated shared CpGs are uniquely mapped to 23 genes and we found these genes are significantly enriched in Kyoto Encyclopedia of Genes and Genomes (KEGG) pathway “Regulation of actin cytoskeleton” (hypergeometric test *P* < 0.05). This observation suggests that the actin cytoskeleton pathway might be affected in both psoriatic and aged skins, which appears compatible with previous findings that aberrant actin cytoskeleton organization may induce Ps [33], and that cytoskeletal integrity declines as a function of age [34]. However, no age-associated CpG is shared between the two comparisons. Likewise, among the 588 age-associated CpGs in PN, 4 CpGs (cg04154027, cg09655403, cg15197065, and cg23005885) were differentially methylated between PP and PN. The difference in the overlaps is again likely due to different sample sizes. By further comparing with the 353 clock CpGs defined by Horvath [18, 25], we interestingly observed 2 clock CpGs (cg10266490 and cg22171829) also differentially methylated between PP and PN.

We finally asked which CpGs have changed their age-association patterns upon developing Ps (or Ps-dependent age-associated CpGs), such as from age-associated to unassociated, or vice versa. To address this, we used a method that resembles Fisher’s transformation (details are described in the “Materials and methods” section) to identify possible Ps-dependent age-associated CpGs. By contrasting PP with PN and NN, respectively, we obtained 301 and 881 CpGs (*P* < 0.01; Additional file 7: Table S6 and Additional file 8: Table S7), but none of these sites passed the threshold of FDR < 0.05. Overall, this data suggests that the age-associated pattern of DNAm in the skin might be largely independent of Ps.

This study is the first to explore whether psoriatic skin tissues suffer from an accelerated DNAm age. Our study is partially motivated by the fact that keratinocytes of Ps involved skins usually undergo abnormal and rapid proliferation. This is a feature that resembles aged individuals and tumors where cells have experienced excessive mitotic cell division. Such excessive division in soma cells could cause widespread passive DNAm change, especially in late DNA replicating domains [35, 36]. This provides us a tempting hypothesis that the involved skins may have an affected DNA methylome and methylation age that resembles the pattern in aged cells or tumor cells. However, our observations, the DNAm age and the age-association pattern of the majority of CpGs appear unaffected by Ps, contradict this hypothesis. This suggests a phenomenon where the methylome of Ps skin cells is affected in a manner that is generally independent to the usual pattern of passive epigenetic drift. As a comparison, the exposure to sun/UV is linked to change in methylome that resembles the aging process [37]. Further investigation into the interactions between Ps and environmental stimuli that are likely important in skin aging might be meaningful, such as UV radiation.

From the technical perspective, DNAm age is calculated through averaging the information of 353 CpGs scattered across the genome, each CpG has very limited weight in the algorithm. Therefore, it is possible that changing the methylation of only a few CpGs, e.g., two clock CpGs are differentially methylated between PP and PN, may not have perceptible impact on the outcome. It may also be that the effect of Ps on aging is relatively small for detection. If this is the case, a larger sample size might be needed to further verify our observation, since our analyses were based on a relatively small set of Ps patients that were exclusively from the Chinese Han population.

Overall, we showed that the multi-tissue DNAm clock model works well in skin tissue of the Chinese Han population. No significant alteration in DNAm age was observed in the uninvolved and involved psoriatic skin tissues, and there is a lack of association between PASI score and age acceleration residual. Therefore, the increase in keratinocyte proliferation and alteration in DNAm caused by Ps may not affect the biological age of psoriatic skin tissue. Our findings expand the understanding of Ps in terms of its impact on skin aging.

## Materials and methods

### Study samples and dataset description

Our previous epigenome-wide association study collected 114 PP and 41 PN from Ps patients (41 patients with paired PP and PN), and 62 NN from health controls from the outpatient at Department of Dermatology, the First Affiliated Hospital, Anhui Medical University, Anhui, China. The detailed inclusion and exclusion criteria of Ps patients and normal controls, the requirement of skin tissues, and methylation experiment were described in our previous study [29]. Here, we analyzed the DNAm dataset of the tissue samples. The clinical features of NN, PN, and PP samples were matched, and no significant difference was detected (*P* > 0.01, Table 1).

### Calculation of DNA methylation age

Horvath [18] developed an epigenetic clock that incorporates the methylation of 353 CpGs, which were obtained through an elastic-net regression, to estimate age. The approach has shown to be robust across a range of human tissues. Here, we took advantage of the corresponding online tool “DNA methylation age calculator” (https://dnamage.genetics.ucla.edu) to estimate the DNAm age of Ps and non-Ps skin samples. Specifically, we prepared and uploaded a compressed text file that include methylation beta values which were measured from all available samples, as well as a file with brief annotation information. After online analysis, an output file with calculated methylation age and other estimates was obtained. The output contains DNAm age and age acceleration residual for each sample. The age acceleration residual is defined as the residual of a linear model where the independent variable is chronological age and the response is DNAm age. As a measure of increase/decrease of DNAm age, the advantage of the residual over the difference between DNAm age and chronological is that the latter could be naturally negatively correlated with chronological age.

### Identification of Ps-dependent age-associated CpG sites

Ps-dependent age-associated CpG sites are considered those age-associated in NN/PN but becoming unassociated in Ps, or vice versa. To obtain them, we first applied a linear regression to test the association between age and CpG methylation for NN, PN, and PP, separately. For the linear model, the independent variable is age and the response is the logit of methylation level *M*, or $\log \frac{M}{1-M}$. For each site, the regression model yielded a coefficient value as well as a corresponding standard error (i.e., *b* and *se*) of the independent variable age. Then, to test the difference of the coefficient between Ps and NN/PN groups, we calculated a *Z* score by using the following equation:

$$ Z=\frac{b_1-{b}_2}{\sqrt{se_1^2+{se}_2^2\ }} $$

Here, *b*_*1*_ and *se*_*1*_ denote the coefficient and the corresponding standard error in Ps, while *b*_*2*_ and *se*_*2*_ denote those in NN/PN. The *Z* score was considered normally distributed, and a *P* value could be obtained for each CpG accordingly.

### KEGG pathway analysis

KEGG pathway analysis was conducted by R package clusterProfiler [38]. For age-associated CpGs, we dropped those located in intergenic regions. We then mapped CpGs to genomic context according to Illumina 450 K methylation array annotation file and CpGs within 5′UTR to 3′UTR for one gene were selected. These genes were searched against the human pathway background. Enrichment test was based on hypergeometric distribution.

### Statistical analysis

Statistical analyses were performed in R software (version 3.3.2) and SPSS (version 16.0). Quantitative data are presented as mean ± standard error. Differences between two groups were analyzed by the Student’s *t* test or Wilcoxon rank sum test. Differences among multiple groups were analyzed by the analysis of variance (ANOVA). *P* values were determined by ANOVA for all quantifications. *P* values < 0.05 were regarded as statistically significant.

## Additional files

Additional file 1: **Table S1.** One hundred CpGs significantly associated with age in NN. (XLSX 14 kb)

Additional file 2: **Table S2.** Five hundred and eighty-eight CpGs significantly associated with age in PN. (XLSX 46 kb)

Additional file 3: **Table S3.** Eight thousand and thirty-two CpGs significantly associated with age in PP. (XLSX 534 kb)

Additional file 4: **Figure S1.** Extra comparisons in addition to Fig. 3b, c. (TIFF 903 kb)

Additional file 5: **Table S4.** CpGs associated with age (FDR < 0.05) in paired PN and PP samples. (XLSX 90 kb)

Additional file 6: **Table S5.** Thirty nine CpGs that are both age-associated in PP and differently methylated between PP and PN. (XLSX 10 kb)

Additional file 7: **Table S6.** Three hundred and one candidates Ps-dependent age-associated CpGs contrasted by PN and PP using a loose cut-off (*P* < 0.01). (XLSX 79 kb)

Additional file 8: **Table S7.** Eight hundred and eighty-one candidates Ps-dependent age-associated CpGs contrasted by NN and PP using a loose cut-off (*P* < 0.01). (XLSX 208 kb)

## Abbreviations

ANOVA: Analysis of variance
BMI: Body mass index
DNAm: DNA methylation
FDR: False discovery rate
KEGG: Kyoto Encyclopedia of Genes and Genomes
NN: Normal skin tissue of health control
PASI: Psoriasis area and severity index
PN: Uninvolved psoriatic skin tissue
PP: Involved psoriatic skin tissue
Ps: Psoriasis
UV: Ultraviolet
