# Supplementary figures and images for: DNA methylation age is not affected in psoriatic skin tissue
Source: Clin Epigenetics. 2018 Dec 27;10:160. doi: 10.1186/s13148-018-0584-y (PMC6307188; doi:10.1186/s13148-018-0584-y)

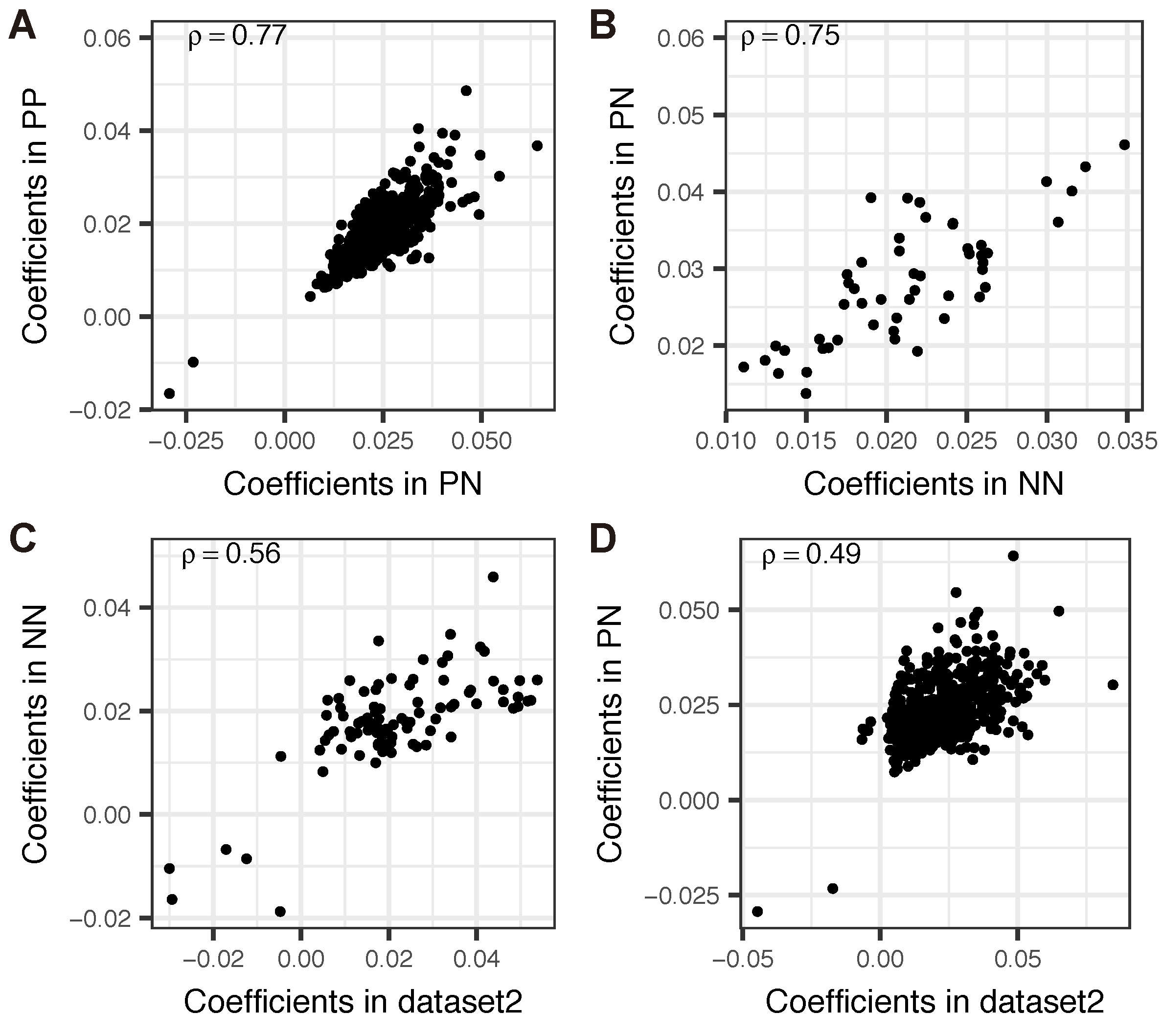

Supplement: Supplementary file 4 — Figure S1. Extra comparisons in addition to Fig. 3b, c. (TIFF 903 kb) [file 13148_2018_584_MOESM4_ESM.tiff]
